# Engineering enzyme catalysis: an inverse approach

**DOI:** 10.1042/BSR20181107

**Published:** 2019-02-12

**Authors:** Clare F. Megarity

**Affiliations:** Department of Chemistry, University of Oxford, South Parks Road, Oxford OX1 3QR, U.K.

**Keywords:** Enzymes, Polyamine oxidase, Reverse Engineering, Stereospecificity

## Abstract

Enzymes’ inherent chirality confers their exquisite enantiomeric specificity and makes their use as green alternatives to chiral metal complexes or chiral organocatalysts invaluable to the fine chemical industry. The most prevalent way to alter enzyme activity in terms of regioselectivity and stereoselectivity for both industry and fundamental research is to engineer the enzyme. In a recent article by Keinänen et al., published in Bioscience Reports 2018, ‘Controlling the regioselectivity and stereoselectivity of FAD-dependent polyamine oxidases with the use of amine-attached guide molecules as conformational modulators’, an inverse approach was presented that focuses on the manipulation of the enzyme substrate rather than the enzyme. This approach not only uncovered dormant enantioselectivity in related enzymes but allowed for its control by the use of guide molecules simply added to the reaction solution or covalently linked to an achiral scaffold molecule.

In this study, flavo-enzymes that catalyse reactions involved in polyamine metabolism were investigated, namely human acetylpolyamine oxidase (APAO), human spermine oxidase (SMOX) and yeast polyamine oxidase (Fms1) [1]. Malfunction in polyamine metabolism is implicated in diseases such as cancer and diabetes [1]; also, it differs between bacteria, parasites and the host organism identifying it as a possible target pathway for novel drugs [2]. It is therefore useful and worthwhile to study the mechanism of these enzymes. The research presented by Keinänen et al. is additionally valuable in terms of enzyme-catalysis for organic synthesis, and it informs at a fundamental level in terms of enzyme structure, catalytic mechanism and selectivity.

Enzyme research that focuses on their potential biocatalytic applications predominantly involves the use of recombinant enzymes, bypassing integral studies *in vivo*. Here is an exemplar holistic approach in which an observation arising from *in vivo* work using transgenic rats [3,4] led to the fundamental enzyme study presented. It was observed *in vivo* that metabolically stable methylated analogues of the enzymes’ natural substrates were metabolised and also that benzaldehyde allowed for the metabolism of methyl-spermidine (MeSpd) [4,5]. The natural substrates for APAO are N^1^ -acetylspermidine, N^1^ -acetylspermine and N^1^, N^12^-diacetylspermine, all of which are in fact achiral [1,6]. The results from the *in vivo* study inspired the authors to initially investigate the activity of APAO with different substrate analogues that were chiral and also the effect of aldehydes on the reaction. Surprisingly, APAO exhibited stereospecificity strongly favouring the (*R*) enantiomer of 1-methylspermidine with a comparable activity to that with its natural substrate, N^1^-acetylspermidine. Moreover, the presence of different aldehydes induced a controllable stereospecificity. It was previously established that benzaldehyde allows APAO to accommodate non-acetylated spermine and spermidine, most likely by the formation of a Schiff base with the polyamines therefore resembling the structure and charge distribution of the acetylated versions [5,6]. Supplementation with benzaldehyde caused APAO to favour the (*R*)-enantiomer of α-methyl spermidine whereas pyridoxal caused the selectivity to change, favouring the (*S*)-enantiomer [6].

In their current paper, this work was extended to include not only aldehyde supplementation but also its covalent attachment to the achiral polyamine substrates. Using the flavin enzymes APAO, Fms1 and SMOX, the authors showed that the aldehyde guide molecules, whether attached covalently or added to the reaction mixture, regulated the enzymes’ stereospecificity and regioselectivity. The Schiff base intermediate formed *in situ* was mimicked to form chemically stable analogues of the N^1^-acetylated derivatives of 1-MeSpd. As for the case when aldehyde was included in solution, APAO favoured the (*R*) enantiomers of these derivatives. It exhibited very low activity with the corresponding (*S*) enantiomers though it is worth noting that its *K*_m_ for these remained very low that suggests they may be efficient competitive inhibitors; this finding could be exploited as an innovative approach to inhibitor design.

A comparison of specificity constant ratios for (*R*):(*S*) enantiomers of N-Ac-Spd shows that APAO ‘prefers’ the (*R*) enantiomer approximately 13 times more than the (*S*) enantiomer whereas when the Schiff base was formed with a bulky aldehyde, the preference for (*R*) was over 100 times that for (*S*). This explained why Schiff bases formed by bulky aldehydes caused almost complete catalysis of one enantiomer favoured over the other. This approach could be used to test the limits of plasticity in both an enzyme’s active site and globally.

Substrate properties were also examined for Fms and it was observed that its reaction with Spd was expectedly very slow and gave three products rather than the expected two, indicating that there were two cleavage sites at exo and endo-N^4^- sites on the spd molecule. The effect of aldehydes on *V*_max_ was less pronounced cf. APAO but they did affect *K*_m_, lowering it significantly. Their most notable effect was on the preferred cleavage site, a change in aldehyde caused the enzyme to favour one site over the other, thus providing a means to control regioselectivity.

Structure-based rational design, directed evolution or a combination of both are the approaches taken to engineer an enzyme [7–13]. Numerous successful examples where enzyme properties have been altered or improved now exist: changing cofactor specificity, swopping enantiomeric preference, increasing resilience to organic solvents, increasing thermal stability, changing the substrate specificity and also decreasing it to make the enzyme more promiscuous, stabilization at specific pH values, and combinations of these [7–13].

A contrariwise approach has been adopted by Keinänen et al., where the focus is shifted away from engineering the enzyme itself and focuses on the manipulation of its substrate both *in situ* and by covalent pre-attachment of the guide moiety. In this way, they were able to not only alter the enzyme’s substrate specificity (the use of benzaldehyde to form a Schiff base with the disallowed, non-acetylated substrates), but also revealed hidden enantioselectivity that was controllable. The inherent enantioselectivity of enzymes is their most exploitable property in the synthesis of fine chemicals and enantiomers of pharmaceutically active molecules that can give rise to different responses. The most renowned example of this is Thalidomide where only the (*R*)-enantiomer gave the desired effect while the (*S*)-enantiomer had a teratogenic effect [14]. The requirement for both enantiomerically pure biologically active compounds, intermediates and other chemicals is paramount and therefore enzyme catalysis is now firmly established in synthetic organic chemistry [9,14–20]. The quest for enzymes with high enantioselectivity better than the corresponding industrial process drives scientists to engineer available enzymes [9,12,16,17]. Keinänen et al. have discovered a previously concealed enzyme stereospecificity that not only could be exploited by industry, but also may point to the possibility of more enzymes which may exhibit this phenomenon. Their guide molecule approach has the potential to be utilized as a probe to unearth other enzymes with latent high enantioselectivity increasing the tool kit for enantiopure synthesis.

Focusing attention on the substrate rather than the enzyme is not a new concept [21–24]. Most examples involve chemical modification of the substrate for example, the addition of a specific functional group such as a docking/protecting group to allow the compound to be more easily accommodated by the enzyme and also to protect against unwanted side reactions [21]. Other examples include variation of the leaving alcohol group on (*R*,*S*)-mandelates for enhanced enantioselecitivity in their hydrolysis [24], the attachment of a removable aryl or alkyl group to an acceptor sugar substrate for α-1-4-galactosyltransferase resulting in a broadening of substrate specificity for the wild-type enzyme [23] and using a ρ-toluenesulfonyl group to block the C-6 position on a glucose moiety altering the specificity of two glucosyltransferases [22]. These examples involve manipulation of the substrate by covalently linking a functional or blocking group, in other words extra steps in the substrate synthesis; Keinänen et al. have shown that for some cases this may be sidestepped by forming a new substrate *in situ*, resembling the structure and charge distribution of the original.

The concept of *in situ* formation of a new substrate could be exploited further. Specifically, there is potential to manipulate enzyme catalysed reactions that involve a Schiff base substrate or intermediate, for example, catalysis by imine reductases [25,26]. The substrate for an imine reductase is an imine (Schiff base); the natural substrates for the polyamine oxidases studied by Keinänen et al*.* were acetylated polyamines yet non-acetylated structurally similar analogues were accommodated by the enzyme in the presence of aldehyde because the Schiff base formed *in situ* resembled the structure and charge distribution of the acetylated polyamine – could this concept be exploited for imine reductase catalysis? Non-imine substrate analogues with perhaps different functional groups and/or stereocentres could be tolerated by the enzyme in the presence of aldehydes, by *in situ* formation of a Schiff base.

Furthermore, enzymes with pyridoxal 5’-phosphate (PLP) as their cofactor catalyse a range of reactions including racemization of amino acids, decarboxylation, retro-aldol and retro-Claisen reactions and transamination reactions [27]. Could such PLP-dependent enzymes be prepared in their apo form and the guide molecule approach used to not only restore activity by using pyridoxal as a guide molecule, but also introduce novel chemistry by the use of different guide aldehydes as replacement analogues for the PLP? This is a template or jigsaw approach whereby a substrate or substrate analogue cannot bind in an active site in the desired orientation unless another piece of the jigsaw is also bound (in this case PLP) to result in a complete and complimentary binding pocket for the substrate. This principle was observed with the enzyme bilvirdin-Ixα reductase that uses the nicotinamide cofactors, NADPH and NADH [28]. The enzyme’s activity with NADH was significantly increased by the addition of inorganic phosphate ions that mimicked the 2’-phosphate of NADPH, docking in its binding pocket in the active site that subsequently allowed NADH to bind in a more stable configuration. Similarly, the NAD-dependent methylenetetrahydrofolate dehydrogenase-cyclohydrolase uses inorganic phosphate ions (along with magnesium ions) to adapt an NADPH-binding site such that it can bind NADH [29]. To inactivate an enzyme by removal of its cofactor, only to reactivate it *in situ* may seem counterintuitive but this approach may lead to interesting observations both in terms of enzyme mechanism and novel chemistry of use to industry. This approach led to a deeper understanding of the complex assembly of the di-iron active site in an apo-hydrogenase [30] and in a similar counterintuitive approach, subtilisin was engineered to be inactive and its activity restored using substrates that contained the missing catalytic group [31].

The guide molecule approach by Keinänen et al. has allowed for the wild type, native state conformational landscape to be surveyed without altering the enzyme’s structure. The native state ensemble consists of conformers that differ, at the very least, in side chain geometries. If the energy landscape is rough, the ensemble contains many different conformations [32] and greater degrees of flexibility give rise to a more extensive ensemble of conformers [33]. As catalysis progresses, the conformer populations within the ensemble change such that catalysis proceeds along a preferred pathway [34]. By using different guide molecules to impose conformational restrictions on substrate molecules, Keinänen et al. have controlled enantioselectivity and regioselectivity by steering the enzyme ensemble towards different conformational landscapes.
